# Crosstalk Between Autophagy and Paraptosis: A New Frontier in Cancer Therapy

**DOI:** 10.3390/ijms27052234

**Published:** 2026-02-27

**Authors:** Sweata Hanson, Deiviga Murugan, Palli V. Jinsha, Anupama Binoy, Bipin G. Nair, Nandita Mishra

**Affiliations:** 1 School of Biotechnology, Amrita Vishwa Vidyapeetham, Kollam 690525, Kerala, India; 2 Department of Biomedical Sciences, Ohio Musculoskeletal and Neurological Institute (OMNI), Ohio University, Athen, OH 45701, USA

**Keywords:** cancer, paraptosis, autophagy, crosstalk, ER stress, mitochondrial dysfunction

## Abstract

Autophagy and paraptosis are two distinct physiological mechanisms involved in regulating cell fate in cancer. Recent studies have demonstrated that autophagy is a crucial process for maintaining cellular homeostasis by facilitating the removal of misfolded proteins and damaged organelles. However, autophagy is found to play a dual role in cancer. Severe ER and mitochondrial dysfunction can trigger different forms of programmed cell death, including autophagic cell death. In cancer cells that evade apoptosis, paraptosis, a caspase-independent alternate death pathway, is triggered by ER and mitochondrial swelling, leading to extensive cytoplasmic vacuolation. It can be induced by natural compounds, metallic complexes, nanoparticles, or chemotherapeutic agents, primarily through excessive ROS production and disruption of protein, thiol, and calcium/ion homeostasis. Autophagy and paraptosis have been found to be connected through crosstalk. While MAPK activation drives paraptosis, ER stress and the unfolded protein response (UPR) can initiate both paraptosis and autophagy. UPR-mediated PERK activation promotes survival autophagy in ER-stressed melanoma, whereas PERK elimination triggers paraptosis via sec61β with unresolved ER stress. Similarly, CHOP and DDIT4 can enhance ER stress and proteotoxicity, thereby favouring paraptosis. This review is unique in exploring the dynamic interplay between autophagy and paraptosis in cancer cells, highlighting promising therapeutic targets for chemotherapy-resistant cancers.

## 1. Introduction

The fate of living cells is regulated by an intricate balance of many complex processes through such signals as DNA damage, protein metabolism, autophagy, growth factors, various developmental cues, and programmed cell death (PCD), typically involving apoptosis [1]. Though these processes remain indispensable for the normal development and maintenance of the living system, they get disrupted in pathological conditions such as cancer, favouring tumorigenesis. Apoptosis serves as an important anticancer strategy due to its specific targets, including anti-apoptotic Bcl-2 family proteins, pro-apoptotic proteins Bax and Bak, and tumour suppressor TP53 [2]. However, cancer cells evade such apoptotic mechanisms by circumventing the signals from these regulatory proteins, thus opening a new avenue for alternative forms of death, like ferroptosis, necroptosis, pyroptosis, etc., in targeting cancer [3].

Paraptosis is a distinct, caspase-independent alternative cell death mode characterized by extensive cytoplasmic vacuolation along with endoplasmic reticulum (ER) and mitochondrial swelling [4]. Over the last two decades, growing research has highlighted the potential of paraptosis in targeting various cancers [5,6,7]. Notably, several paraptosis inducers have also demonstrated a role in sensitizing cancer to chemotherapeutic drugs, thereby overcoming drug resistance. The essential modulators of paraptosis and its emerging role in tumour biology have been explained in various reviews [5,8,9,10].

Autophagy is a key homeostatic mechanism that involves the lysosomal degradation of dysfunctional cellular components via the formation of double-membraned autophagosomes. Though autophagy was initially classified as a programmed cell death mechanism (type II) [11], the role of autophagy in survival has also been demonstrated widely [12,13,14,15]. Various proteins like autophagy-related genes (ATGs), beclin 1, mammalian target of rapamycin (mTOR), etc., are the key regulatory proteins known to activate autophagy [16]. In cancer, autophagy exhibits a dual role, either by accelerating tumour progression or suppressing tumorigenesis by alterations in these regulatory proteins [15].

Several studies have explored and extensively studied the crosstalk with autophagy and different cell death pathways like ferroptosis [17], apoptosis [18], necroptosis [19], etc., in cancer. However, despite the growing recognition of paraptosis as a potential strategy to overcome chemoresistance and reported co-occurrence of both paraptosis and autophagy in cancer cells [20,21,22,23,24,25,26,27], the interplay between autophagy and paraptosis is not yet emphasized. This review explores the crosstalk between paraptosis and autophagy during cancer therapy, leading to the identification of key proteins that link these pathways, to support novel cancer therapeutic strategies.

## 2. Autophagy: The Degradation and Recycling Machinery

**Figure 1:**
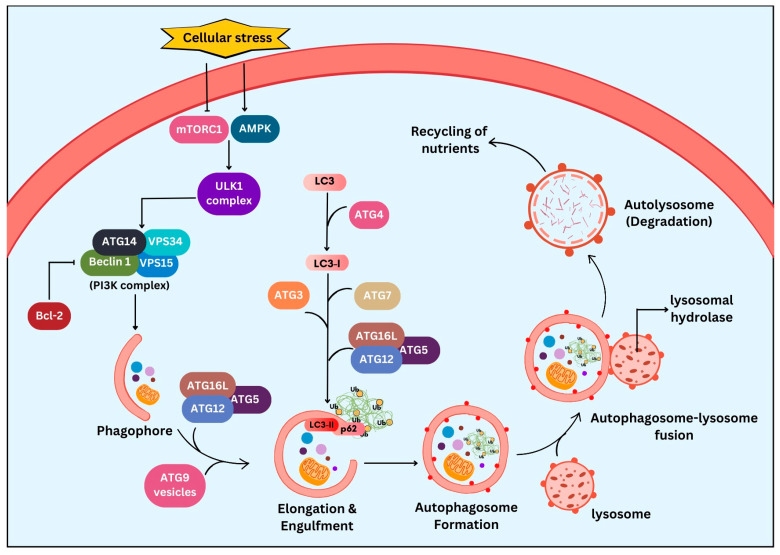
Molecular mechanism of autophagy. Major steps in the activation of autophagy, which include nucleation, phagophore formation, elongation and cargo sequestration, autophagosome formation, and autophagosome–lysosome fusion leading to degradation and recycling. mTORC1 (mammalian target of rapamycin complex 1); AMPK (adenosine monophosphate-activated protein kinase); ULK1 (Unc 51-like autophagy-activating kinase); ATGs (autophagy-related genes); VPS 34/15 (vacuolar protein sorting 34 and 15); LC3 (microtubule-associated protein 1A/1B light chain 3); PI3K (phosphatidylinositol 3-kinase complex); Bcl-2 (B-cell lymphoma 2). This figure was generated using Canva (www.canva.com, accessed on 13 December 2025).

The advent of autophagy (Greek ‘auto’ means self, ‘phagy’ means to eat) dates to the 1960s. The delivery of non-functional intracellular content to lysosomal digestion was termed ‘autophagy’ by de Duve in 1963. The importance of autophagy in cellular survival is widely recognised, particularly under nutrient starvation conditions. Similarly, the involvement of autophagy in cancer therapy is being well explored [28]. The autophagy machinery aims to deliver the autophagic substrates like aggregated proteins and damaged organelles to the lysosomes for degradation by forming double-membraned autophagosomes to recycle and reuse the cellular components. The basic process of autophagy has been well conserved in species as diverse as plants, flies, yeast, and mammals, as found in ATGs [29]. As shown in Figure 1, autophagy is initiated in response to various cellular stresses like nutrient or energy deprivation or in the presence of damaged organelles or misfolded protein aggregates. The activation of AMPK (adenosine monophosphate-activated protein kinase) and inhibition of mTORC1 (mammalian target of rapamycin complex 1) [30] leads to the formation of a pre-initiation complex, which is the ULK (Unc 51-like autophagy-activating kinase) complex. The pre-initiation complex in turn activates the initiation complex, the class III PI3K (phosphatidylinositol 3-kinase) complex comprising beclin 1, ATG14, VPS34, and VPS15 (vacuolar protein sorting 34 and 15). Phagophore formation is then initiated with the dissociation of Bcl-2 protein from beclin 1 and the activation of AMPK. The subsequent elongation of the phagophore is assisted by two ubiquitin-like protein conjugation systems, the ATG12–ATG5–ATG16L complex and ATG4/LC3 (microtubule-associated protein 1A/1B light chain 3) system, resulting in autophagosome formation. This autophagosome formation requires the conjugation of LC3 with phosphatidylethanolamine (PE), allowing the incorporation of LC3 into the autophagosomal membrane. ATG4-dependent proteolytic cleavage of pro-LC3 to LC3-I is followed by the conjugation of phosphatidylethanolamine with LC3-I, converting it to LC3-II. The action of ATG7, ATG3, the ATG12–ATG5–ATG16L complex, and membrane recruitment protein ATG9 stably associates the LC3-II with the autophagosomal membrane. Sequestosome 1 (SQSTM1/p62), a ubiquitin-binding protein, plays an important role in cargo identification for selective degradation by acting as an adaptor protein that interacts with LC3-II to target the protein aggregates for autophagic degradation. The fusion of lysosome with autophagosome forms the autolysosome. The sequestered autophagic substrates are degraded in the autolysosome and are released into the cytosol for recycling [31]. Autophagy can be broadly categorized into non-selective form (no ubiquitination), which typically occurs during nutrient starvation and involves bulk degradation with energy production and selective forms (cargo ubiquitination), where the process can be highly specific to certain organelles, such as peroxisomes [32], mitochondria [33], and the endoplasmic reticulum [34], among many, for cellular maintenance and homeostasis [35].

It is possible to understand this conventional process as ‘survival autophagy’, but its role in cancer is two-faced [36,37]. Autophagy acts as a tumour suppressor in the early stages of carcinogenesis by degrading damaged mitochondria and peroxisomes, preventing ROS accumulation and DNA damage. On the other hand, it supports tumour cell survival at advanced stages by alleviating stress in the tumour microenvironment [38]. The involvement of autophagy in cancer can be attributed to mutations in tumour suppressor genes or oncogenes. The first study on the association between autophagy and cancer showed lower BECN1 (beclin 1) expression and autophagic activity in breast cancer when compared to normal breast epithelia [39]. With further research, it was shown that cells expressing oncogenes such as KRAS (Kirsten rat sarcoma virus) required autophagy for tumorigenesis [40]. To understand these paradoxical effects of autophagy, it is important to consider several individual factors, such as the type, stage, or molecular nature of cancer [14,41].

The regulation of autophagic activity in cancer pathogenesis is essential, owing to the dynamic nature of autophagy. mTORC1, a key regulator of autophagy, is known to be active in cancer cells. Thus, the activation of PI3K/Akt/mTORC1 inhibits autophagy while promoting tumour growth [30]. Similarly, the tumour suppressor gene TP53 regulates mTORC1 signalling proteins such as tuberous sclerosis complex 2 (TSC2) and AMPK, as well as autophagy genes such as ULK1, ULK2, and atg7, to promote autophagy [42]. Apart from cancer development, autophagy is also thought to play a role in cancer metastasis. Neighbour of BRCA1 gene 1 (NBR1), a homologue of p62, accumulates inside the cell and promotes metastasis [43].

Autophagy acts as a backup mechanism for different cell death pathways that focus on reducing cellular stress to maintain homeostasis in the body. Various proteins have been highlighted in different studies to act as a link in different cell death pathways and autophagy, for example, beclin 1 and p53 acting as an internode in connecting apoptosis and autophagy, sirtuin 2 (SIRT2) in linking necroptosis and autophagy [18], and mTOR in regulating ferroptosis and autophagy [17,44]. Although the interplay between autophagy and different cell death pathways can be complex and context-dependent, it is crucial to understand the dynamic links between these processes. Elucidating how autophagy is regulated alongside other cell death pathways may reveal novel therapeutic strategies for the development of effective cancer therapies.

## 3. Paraptosis: An Alternate Cell Death Pathway

Paraptosis is a novel alternative programmed cell death pathway reported by Bredesen and his group [4]. Paraptosis is found to play a vital role in different disease conditions like neurodegenerative diseases [45], glaucoma [46,47], and cancer [48,49]. Paraptosis shows characteristic morphological changes like the formation of extensive cytoplasmic vacuolation exhibiting swelling of ER and/or mitochondria, absence of DNA fragmentation, and cell death independent of caspase cleavage, unlike apoptosis [4]. Its ability to combat chemoresistant cancer cells makes paraptosis an important form of cell death [50]. Different inducers like natural compounds [51,52], metallic complexes [53], nanoparticles [54], photosensitisers [55], etc., have been found to cause paraptotic cell death in cancer cells. The underlying mechanisms inducing paraptosis include insulin-like growth factor 1 receptor (IGF1R) activation [4], calcium signalling [56], MAPK activation [57], ER stress via proteasomal inhibition [58], ROS generation [59,60], the opening of ion channels [61], etc. Activation of paraptosis can be cell type- and context-specific in various types of cancer. Proteins identified to play a crucial role in paraptosis regulation include, but are not limited to, ALG-2-interacting protein 1 (AIP1/Alix) [62], ubiquitin-specific protease 10 (USP10) [63], phosphatidylethanolamine-binding protein 1 (PEBP-1), prohibitin (PHB) [64], thioredoxin reductase 1 (TrxR1) [65], Src homology 2-containing protein tyrosine phosphatase 2 (SHP2) [23], valosin-containing protein (VCP) [66], and thyroid hormone receptor interacting protein 13 (TRIP13) [67]. The autophagy marker proteins SQSTM1/p62 and MAP1LC3B are upregulated during paraptosis in cancer cells [48,49], but their probable role in inducing paraptosis has not been elucidated for long. Recent evidence suggests a connection between p62 and paraptosis via aggresome accumulation [26] and inhibition of autophagy [22,23,25].

IGF1R is a cell surface receptor actively involved in cell development and proliferation. This receptor was initially reported to have a dynamic effect in inducing paraptosis in 293T cells and mouse embryonic fibroblast cells [4]. Later, it was found that IGF1R intracellular domain (IGF1R-IC)-induced paraptosis was mediated by MAPK activation, highlighting the importance of its kinase activity [52,62]. Other receptors, such as vanilloid receptor subtype 1 (VR1), TAJ/TROY, neurokinin 1 receptor (NK1R), epidermal growth factor receptor (EGFR), 1-nitropyrene (1-NP), etc., are known to induce paraptosis in cancer cells [8]. Moreover, the critical role of MAPK in paraptosis has been elaborately studied in drug-resistant ovarian cancer cells through the CRISPR/Cas9 technique, highlighting the role of SHP2 protein, an upstream regulator of MAPK in paraptosis induction [23]. In addition, AIP1/Alix was identified to inhibit the activation of MAPK, thereby hindering paraptotic cell death [62]. PHB and PEBP-1 are other vital proteins identified as mediators and inhibitor of paraptosis [64]. This alternative cell death mode requires new protein synthesis for its cytotoxic effect, as evidenced by pretreatment with cycloheximide (CHX), a protein synthesis inhibitor [68,69]. MAPK, mTORC1, and Akt, the central regulators of protein synthesis, have been found to play important roles in paraptosis induction [23,27,66,70,71,72,73]. The endoplasmic reticulum is the site for protein synthesis, folding and sorting, and calcium sequestration. During paraptosis, the newly synthesised proteins accumulated in the ER are often misfolded due to the higher load of protein and proteasomal inhibition [65] or improper folding by chaperones like binding of immunoglobin protein (Bip). These misfolded proteins can trigger ER stress and UPR, directing the synthesis of more chaperones required for folding as well as inhibition of global translation [68].

UPR gets activated due to prolonged stress in the ER, which disrupts cellular homeostasis. To normalise this stress condition, UPR is initiated, where three ER membrane proteins—PKR-like ER kinase (PERK), inositol-requiring enzyme 1α (IRE1α), and activating transcription factor 6 (ATF6)—are activated. In normal conditions, these transmembrane proteins are bound to the ER chaperone, Bip, but during stress conditions, Bip is released from them, leading to their activation [27,74,75]. Studies have also reported the crucial role of ER stress-sensor proteins during paraptosis in various cancer cells [63,69]. Moreover, downregulation of several ER stress and UPR markers like protein disulphide isomerase (PDI) [76], PERK [6], C/EBP homologous protein (CHOP) [77], and ATF4 [59] has been shown to regulate paraptosis in cancer cells.

**Figure 2:**
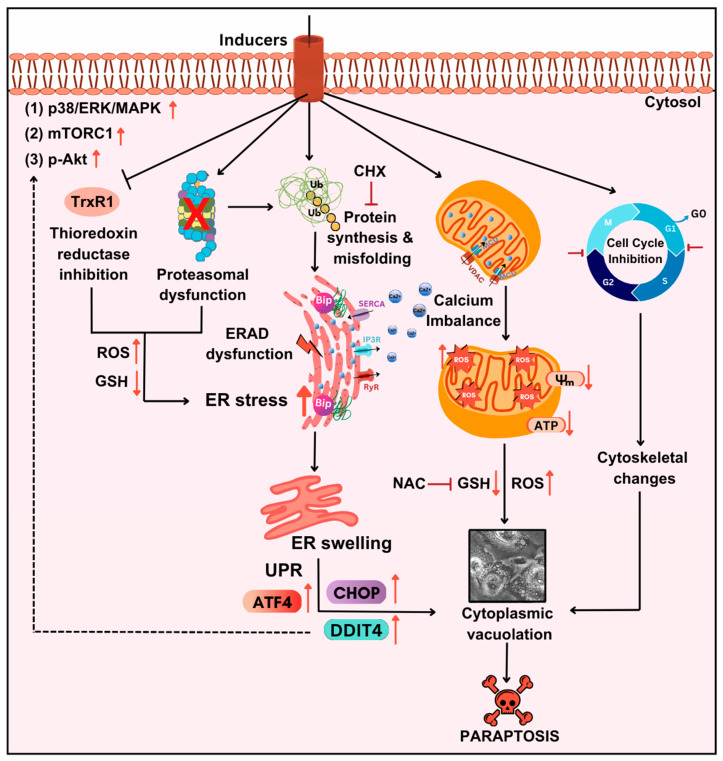
Characteristic features of ER stress-induced paraptosis. Cancer cells, upon treatment with natural products, proteasomal inhibitors, or metallic complexes, activate the MAPK and mTORC1 pathways, leading to increased protein synthesis and misfolding. The misfolded proteins induce ER stress-mediated extensive cytoplasmic vacuolation due to dilation of the ER, followed by unfolded protein response, calcium imbalance, dysfunction of mitochondria, ROS generation, cell cycle inhibition, and cytoskeletal changes resulting in cell death. ERK (extracellular signalling-regulated kinase); MAPK (mitogen activated protein kinase); mTORC1 (mammalian target of rapamycin complex 1); TrxR1 (thioredoxin reductase 1); ROS (reactive oxygen species); GSH (glutathione); ERAD (endoplasmic reticulum-associated degradation); CHX (Cycloheximide); ER (Endoplasmic Reticulum); NAC (N-acetyl cysteine); ATP (adenosine triphosphate); UPR (unfolded protein response); CHOP (C/EBP homologous protein); ATF4 (activating transcription factor 4); DDIT4 (DNA damage-inducible transcript 4); SERCA (sarco/endoplasmic reticulum Ca^2+^-ATPase); IP3R (inositol-1,4,5-triphosphate receptor), RyR (ryanodine receptor); VDAC (voltage-dependent anion channel); MCU (mitochondrial calcium uniporter). This figure was generated using Canva (www.canva.com).

The ER and mitochondria are interconnected via the mitochondria-associated endoplasmic reticulum membrane (MAM), which helps in calcium signalling. Changes in the ER can affect Ca^2+^ homeostasis, thereby increasing cellular stress. As shown in Figure 2, the shuttling of calcium ions between the ER and mitochondria occurs through different channels, such as sarco/endoplasmic reticulum Ca^2+^-ATPase (SERCA), inositol-1,4,5-triphosphate receptor (IP3R), and ryanodine receptor (RyR) in the ER, and voltage-dependent anion channel (VDAC) and mitochondrial calcium uniporter (MCU) in mitochondria [78]. An imbalance in calcium ion homeostasis results in mitochondrial damage contributing to the progression of paraptotic cell death. Studies have also shown that this disruption can impair cellular oxidative metabolism, resulting in the loss of mitochondrial membrane potential (MMP), thus increasing ROS generation [79]. This in turn causes a significant decrease in ATP production, leading to paraptotic cell death [24,51,80,81]. Additionally, many paraptosis-inducing agents influence cell cycle regulation, leading to cell death following cell cycle arrest [82,83]. Although several mechanisms are involved, ER stress plays a crucial role in driving paraptotic cell death. Taking together the importance of ER stress-mediated UPR activation in autophagy and paraptosis, we aimed to correlate how the extent of ER stress influences in determining cell’s fate. The primary characteristic features of autophagy and paraptosis, as outlined in Table 1, would be helpful in identifying two distinct molecular pathways in the cancer cells.

## 4. The Intersection of Autophagy and Paraptosis: Key Players

Autophagy in cancer cells is upregulated under different stress conditions, including extracellular stress such as hypoxia, nutrient deprivation, microbial infection, and intracellular stress induced by the accumulation of misfolded or unfolded proteins, damaged organelles, and high metabolic energy demands. Similarly, paraptosis is also activated in cancer cells under ER stress conditions due to an imbalance in protein homeostasis induced by high growth and cellular changes by external inducers. The type of cancer cell, growth conditions, type of inducer/stimuli, and level of ER stress determine the cell’s fate. Since both autophagy and paraptosis play an essential role in the cells, there is a high possibility of crosstalk between them, enabling co-regulation. Recent reviews highlight multiple coordination of autophagy and paraptosis and the role of the proteins involved in this crosstalk in cancer. An overall understanding of cellular events, with detailed molecular mechanisms of crosstalk, will play a vital role in the successful development of anticancer therapies in the future.

### 4.1. Beclin 1: A Key Autophagic Regulator

Beclin 1, a Bcl-2-interacting protein, is known to play an essential role in autophagy induction. Beclin 1 is encoded by the BECN1 gene, which in its haplodeficient form acts as a tumour suppressor. Allelic loss of this gene is observed in breast, ovarian, and prostate cancers [95]. Beclin 1 is the mammalian autophagy gene homologous to the yeast atg6 gene. Beclin 1 acts as a positive regulator of autophagy, but its interaction with Bcl-2 anti-apoptotic protein can neutralise its pro-autophagic nature [96]. Bcl-2 protein binds to beclin 1 and interferes with the autophagic process, whereas beclin 1 does not affect the anti-apoptotic function of Bcl-2 [97]. Many studies have also reported the increased expression of beclin 1 during autophagy induction in cancer cells [98].

Under normal physiological conditions, Bcl-2 binds to beclin 1 and inhibits autophagy. During nutrient starvation, beclin 1 dissociates from Bcl-2 as a result of phosphorylation of Bcl-2 by c-Jun amino-terminal kinase 1 (JNK1) and interacts with different proteins like VPS34, VPS15, and ATG14 to form a PI3K class III nucleation complex, thus promoting autophagy [99]. Studies have shown that beclin 1 protein levels decrease during ER stress-induced paraptosis in cancer cells. Additionally, knockdown of beclin 1 has been reported to sensitize glioblastoma cells to NIM811-mediated paraptosis [21,27,100]. These findings point to the possibility of beclin 1 being an important player in linking both autophagy and paraptosis.

### 4.2. MAP1LC3B: An Autophagic Marker

MAP1LC3B (microtubule-associated protein one light chain 3B) is a central regulator of autophagy. Nascent LC3B is processed to cytosolic LC3-I by ATG4, which is associated with phosphatidylethanolamine and lipidated to form LC3-II. LC3-II helps in the elongation and maturation of autophagosomes and is considered a marker of autophagy. The autophagosome containing LC3-II and cargo protein is fused with the lysosome to form an autolysosome, where the cargo within is degraded [87]. The level of LC3-II increases during autophagy, allowing it to be used for monitoring autophagosome formation. Many studies have reported a decrease in LC3-I and an increase in LC3-II during autophagy, which marks this protein as an essential autophagic marker [101]. However, in paraptosis, increased expression of LC3-I and LC3-II were observed when cancer cells were exposed to different inducers like small molecules [48,49], radiation [102], metallic complexes [53], etc. Although LC3 is primarily associated with autophagy, its increased accumulation during paraptosis has been reported to be associated with the inhibition of autophagic flux [22,23,25].

### 4.3. SQSTM1/p62: Autophagosome Cargo Protein

SQSTM1, a sequestosome 1 protein also known as p62, is involved in the autophagy system and the ubiquitin–proteasome system, exhibiting its dual role [103]. The p62 protein acts as an autophagy receptor, where its C-terminus binds to the polyubiquitinated cargo proteins and the other end to LC3-II inside the autophagosome [104]. p62 plays a lead role in sequestering the ubiquitinated proteins inside the autophagosome. The interaction between p62 and LC3-II is required to efficiently target autophagosome cargo proteins, which will eventually be degraded by fusion with the lysosome. Thus, the level of p62 expression will be reduced during autophagy [105]. While the role of p62 in autophagy is well established, emerging studies also highlight its significance in paraptosis [48,106,107]. It has been widely explored that proteasomal inhibition accelerates the induction of paraptosis [49,77,108]. Increased accumulation of p62 levels due to proteasomal inhibition is one of the alterations observed during paraptosis. Moreover, studies have reported that knockdown of p62 partially reversed ER stress, cytoplasmic vacuolation, and cell death, specifying the importance of p62 in paraptosis [26]. Recent reports have highlighted that the elevated levels of p62 during paraptosis are due to autophagic flux inhibition [22,23,25].

### 4.4. NBR1: Autophagosome Cargo Protein

NBR1 is a selective autophagy receptor that plays a prominent role in autophagy. NBR1 has a protein-binding domain (PB1 domain), ZZ-type zinc finger, and C-terminal ubiquitin-binding (UBA) domain, which recognises and delivers the ubiquitinated cargo proteins to the autophagosome for degradation after fusion with the lysosome [109]. This can bind to both LC3B and ubiquitinated proteins and process them for degradation. Although p62 and NBR1 form a dimer through their PB1 domain and have a similar role in autophagy, both proteins function independently [110]. NBR1 closely resembles the p62 protein for this reason, and is critical for initiating autophagy [111]. Impaired autophagy in tumour cells results in the accumulation of NBR1 and p62 proteins [43]. Similarly, during paraptosis, these autophagic substrates were elevated after treatment with celastrol in cancer cells [112], which indicates its role in the regulation of both autophagy and paraptosis.

### 4.5. TRIB3: An ER Stress Sensor Protein

TRIB3 (tribbles homologue 3), an ER stress-sensor protein, belongs to the pseudokinase family, and is known for its role as a molecular switch in managing various stress conditions, such as disrupted cellular homeostasis, metabolic disorders, and cancer. It plays a significant role in cellular stress responses. Notably, TRIB3 has been shown to promote MYC-associated lymphoma by inhibiting ubiquitin protein ligase E3B (UBE3B)-mediated degradation of the oncogenic protein MYC [113].

TRIB3 also plays a critical role in autophagy regulation through its interaction with the autophagy adaptor protein p62. This interaction interferes with the binding of p62 and LC3 to ubiquitinated proteins, thereby impairing the degradation process of these proteins [114]. Such disruption in autophagy can lead to the accumulation of oncogenic factors like epidermal growth factor receptor (EGFR), matrix metalloproteinases (MMPs), and c-MYC, which are implicated in tumour metabolism and cellular proliferation [115].

Furthermore, TRIB3 expression is upregulated in a c-MYC-dependent manner, enhancing its interaction with p62 in aggresomes. Protein aggresomes can undergo ubiquitination and be targeted to p62 for degradation and clearance [116]. This interaction has been linked to the induction of paraptosis, a type of programmed cell death, particularly following treatment with a combination of everolimus and ginsenoside in cancer cells [26]. Thus, TRIB3 plays a regulatory role in both autophagy and paraptosis, positioning it as a key modulator of cell fate under stress conditions and a potential target in cancer therapy [117].

### 4.6. PINK1: A Mitophagy Marker Protein

PINK1 (PTEN-induced putative kinase 1), a serine/threonine kinase involved mainly in mitochondrial regulation, has been shown to play an essential role in the selective autophagy (mitophagy) through the regulation of parkin, an E3 ubiquitin ligase. It acts as a vital inducer of mitophagy through the PINK1–parkin pathway and is upregulated in cancers due to a major tumour suppressor gene, PTEN, providing a cancer survival mechanism culminating in cellular resistance to therapies [118]. PINK1 has been reported to have both pro- and antitumorigenic activity, as its expression is decreased in several cancers such as ovarian, liver, and renal carcinomas, whereas it is increased in malignancies including endometrial, parathyroid, and haematological cancers [119]. A study reported the cytoprotective effects of PINK1-dependent mitophagy in cancer cells with the treatment of an E3 ubiquitin ligase, A1RIH1 [120]. Studies with heavy metals like antimony have been shown to promote bladder cancer through inhibition of the PINK1–parkin pathway by modulating mitophagy [121]. With increasing evidence of the importance of PINK1-dependent mitophagy in cancer, the relevance of PINK1 in paraptosis is also studied, where the protein is said to be involved in cytoplasmic vacuolation, suggesting that PINK1-dependent mitophagy pathway plays a critical role in the induction of paraptosis [122].

### 4.7. Alix: ESCRT-Associated Protein

Alix, the multifunctional ALG-2-interacting protein X, plays an important role in various cellular processes like endosomal sorting and autophagy. This protein was initially found to be involved in mediating apoptosis by binding to ALG-2, which is a calcium-binding protein [123]. Alix is described as an endosomal sorting complex required for transport (ESCRT)-associated protein and is also known as PDCD6IP (programmed cell death six-interacting protein). During autophagy, the ATG3–ATG12 complex interacts with Alix and promotes the maturation of autophagosome formation containing the ubiquitinated cargo proteins, which will be degraded after fusion with the lysosome [124]. Studies suggest a crucial role of Alix in autophagy regulation. However, it was reported that the increased expression of AIP1/Alix results in the attenuation of both JNK and MAPK via IGF1R activation, thus blocking paraptosis induction [62]. Several findings pointed out the significant role of Alix as an endogenous inhibitor of paraptosis [21,57,59].

### 4.8. ER Stress and UPR Proteins

The endoplasmic reticulum is a complex organelle that provides the platform for nascent protein synthesis, protein folding, Ca^2+^ storage, and lipid and carbohydrate metabolism [125]. Molecular chaperones within the ER, including GRP78/Bip, lectin chaperones, J-domain proteins, etc., play a major role in maintaining ER homeostasis. Various pathological and physiological conditions cause an imbalance in ER homeostasis, leading to the accumulation of unfolded or misfolded proteins within the ER lumen, causing ER stress. In response to various stress conditions, the ER interacts with other organelles, including mitochondria, for regulating cellular Ca^2+^ homeostasis [126], endosome/Golgi for lipid exchange and metabolism [127], and phagocytes forming autophagosomes, thereby participating in autophagy [128]. For combating ER stress and restoring homeostasis, two major pathways are activated: the UPR and ER-associated degradation (ERAD) [129]. Three ER stress-sensor proteins govern the UPR in mammalian cells, namely IRE1α, PKR-like ER kinase (PERK), and activating transcription factor 6 (ATF6). Normally, these sensor proteins remain bound to a key ER chaperone, HSPA5/GRP78/Bip, preventing its oligomerisation-induced activation. When ER homeostasis is disturbed, these chaperones dissociate from the ER sensor proteins and regulate the activation of a complex ER-to-nucleus signalling pathway via various downstream transcription factors, thus restoring ER homeostasis [130]. During ER stress, IRE1 oligomerises, followed by trans-autophosphorylation and activation of the RNase domain, which causes the splicing of X box-binding protein 1 (XBP1) [131]. Spliced XBP1 (XBP1s) regulates the transcription of genes involved in the ERAD pathway, ER quality control mechanisms, ER/Golgi biogenesis [132], oxidative stress responses, and redox homeostasis [133]. During prolonged activation of ER stress, IRE1α acts as a molecular switch regulating both adaptive and suicide gene response [134]. During ER stress, the IRE1α–JNK pathway is activated, enhancing autophagy as a cell survival mechanism [135]. Activated PERK dissociates from Bip, oligomerises, and is auto-phosphorylated. The phosphorylated PERK then phosphorylates eukaryotic initiation factor 2α (eIF2α), reducing global protein synthesis and decreasing the ER load. Alternatively, by increasing the translation of ATF4, p-eIF2α increases the expression of cytoprotective genes, autophagy-related genes, and ERAD-related genes [136]. ER stress-induced activation of the PERK–eIF2α–ATF4 pathway upregulates the expression of autophagy genes [137]. Besides eIF2α, PERK phosphorylates and activates nuclear factor E2-related factor 2 (Nrf2), a master antioxidant transcription factor maintained in an inactive state through its interaction with Kelch-like Ech-associated protein 1 (KEAP1) [138]. ATF6 is transported to the Golgi in response to ER stress, where it is cleaved by S1 and S2 proteases. The cleaved ATF6 is translocated to the nucleus, where it promotes the production of ER stress response genes and ER chaperones [137]. The expression of death-associated protein kinase 1 (DAPK1) is upregulated by ATF6, which further activates beclin 1 through phosphorylation, resulting in the induction of autophagic machinery [139]. Molecular pathways related to ER stress-induced autophagy include IRE1α–JNK–Bcl–2, PERK–eIF2α–ATF4, and ATF6–XBP1–ATG [140]. Disturbance to ER homeostasis, if not restored by UPR response and autophagy, results in cell death. Both in vitro and *in vivo* studies using the bioactive compound cantharidin (CTD) showed increased mRNA levels of ER stress-regulated proteins (GRP78, ATF4, eIF2α, and CHOP) along with autophagic proteins (LC3, beclin 1, ATG3, and ATG7). The ER stress inhibitor 4PBA inhibits the upregulation of these genes, indicating the role of ER stress in the activation of autophagy [141]. ER stress is one of the mechanisms that induce paraptosis [51]. The elevated expression of ER stress markers during paraptosis has been reported in several studies [58,75,82]. VER155008, an HSP70 inhibitor, induced paraptosis with increased mRNA levels of Bip and CHOP in anaplastic thyroid carcinoma cells [68]. Elaiophylin (a paraptosis-inducing compound) induces ER stress and activates IRE1 along with increased expression of ATF4 and CHOP [23]. Calreticulin is an ER-resident protein that lines cytoplasmic vacuoles formed during ER stress. An IP3 receptor-mediated release of Ca^2+^ from the ER initiates paraptotic cell death in response to ER stress [142]. Epimedokoreanin B (EKB), a prenylated flavonoid-induced paraptosis, showed downregulation of Alix and upregulation of ER stress marker proteins [25]. Photodynamic therapy is also known to induce cytoplasmic vacuolation by the accumulation of ER stress-related proteins [86]. ER stress and UPR signalling play a major role in the crosstalk between autophagy and paraptosis and in the cell’s decision to be or not to be.

### 4.9. Calcium Signalling Regulatory Proteins

Ca^2+^, a secondary messenger, plays a major role in the regulation of cell survival/death processes. It has been reported that disturbances to Ca^2+^ homeostasis result in various modes of cell death in cancer cells via upregulation or downregulation of various regulatory proteins [140]. Under normal physiological conditions, ER and mitochondria mainly regulate calcium homeostasis within a cell. The active transport systems, including the plasma membrane Ca^2+^-ATPases (PMCAs) and SERCAs, RyRs, and IP3Rs, play a predominant role in maintaining the electrochemical gradients for Ca2+ within the cell [143]. The intracellular Ca^2+^-release channels, IP3Rs, and RyRs mediate the calcium release from the intracellular stores, while the ATP-dependent SERCA pump actively transports Ca^2+^ into the ER. This maintains a lower cytosolic Ca^2+^ concentration of close to 0.1 µM compared to ~1 mM and ~0.5 mM concentrations within the extracellular milieu and ER, respectively [144]. The Ca^2+^ ions released from the ER by IP3Rs or RyRs flux across the outer mitochondrial membrane (OMM) and inner mitochondrial membrane through the VDAC [145,146] and the MCU complex [147,148]. A complex antiporter system, including the mitochondrial Na^+^–Ca^2+^ exchanger (mNCX) and the mitochondrial H^+^–Ca^2+^ exchanger (mHCX), plays a major role in mitochondrial Ca^2+^ efflux mechanisms to restore the basal state. Studies have reported that Ca^2+^ signalling is required for mediating autophagy with the involvement of different calcium channels [148]. Proteasomal inhibition causes the accumulation of misfolded proteins within the ER lumen, resulting in ER stress and unfolded protein response. Mitochondria-associated membranes (MAMs), which connect the ER and mitochondria, play a major role in maintaining Ca^2+^ homeostasis [149]. It was also reported that calcium release from the ER to mitochondria plays an important role in inducing paraptosis [123] by causing calcium imbalance in cancer cells [150]. Therefore, calcium signalling is vital in influencing cellular processes like autophagy and paraptosis [100].

### 4.10. Cell Cycle Regulatory Proteins

Cell cycle regulatory proteins such as cyclins, cyclin-dependent kinases (CDKs), and cyclin-dependent kinase inhibitors (CDKIs) are crucial for the orderly progression of the cell cycle [151], and their dysregulation can lead to cancer [152]. The balance between CDKs and CDKIs is critical for cell cycle regulation and autophagy, both of which play significant roles in cancer. Inhibition of CDKs such as CDK4/6 and CDK2 has been shown to induce autophagy, contributing to anticancer effects, particularly in cancers like multiple myeloma [153]. CDKIs, such as p21 and p27, can also influence autophagy by modulating mTORC1 activity, and their upregulation through autophagy has garnered interest in cancer research [154,155,156]. Additionally, p53 plays a dual role in regulating autophagy, promoting it when localised in the nucleus and inhibiting it when present in the cytoplasm [157,158]. Several studies have highlighted the involvement of CDKs and cyclins in cell cycle arrest during paraptosis, demonstrating their regulatory roles in this form of cell death [22,73,83,159]. For example, activation of the CDK7/CDK9–Rbp1 signalling pathway enhances paraptosis by upregulating genes associated with the UPR, ER stress, and heat shock proteins [160]. In contrast, aloperine treatment in glioblastoma cells has been shown to reduce levels of cyclin D1, CDK6, and CDK4, alongside a decrease in cell cycle inhibitors such as p18 and p21 [22]. Additionally, knockdown of CBP or Ku70 in melanoma cells causes S-phase arrest and increases ROS levels due to elevated NADPH-oxidase 2 (NOX2) activity, contributing to paraptotic cell death [160].

### 4.11. Ubiquitin-Specific Peptidase 10 (USP10)

USP10 is a deubiquitinase enzyme that removes ubiquitin tags from target proteins, thereby preventing their degradation. This deubiquitinase (DUB) is reported to play a significant role in modulating different functions, like recycling ubiquitin and maintaining intracellular protein homeostasis. During autophagy, USP10 exhibits its recognising role in binding to beclin 1, a key regulator of autophagy. It has been reported that USP10 removes the ubiquitin tags from beclin 1 that are targeted for degradation, thus promoting autophagy [161]. USP10 has also been found to be expressed in malignant breast cancer cells during paraptosis, thus acting as a novel paraptotic regulator [63]. Though USP10 can act as both a tumour suppressor and oncogene in a context-dependent manner [162], its involvement in both pathways highlights its potential as an important player.

### 4.12. High-Mobility Group Box 1 (HMGB-1)

HMGB-1 acts as a damage-associated molecular pattern (DAMP), a danger signal during inflammation, and its function depends upon cellular localisation. In the nucleus, HMGB-1 binds to the chromatin and targets DNA for cell death, while in the cytoplasm, HMGB-1 can induce autophagy [163]. It has been reported that HMGB-1 can promote autophagy by displacing Bcl-2 from beclin 1, a key inducer of autophagy in response to cellular stress [164]. Moreover, the translocation of HMGB-1 from the nucleus to the cell periphery in T9 glioma cells, acting as a danger signal to stimulate immune response, highlights its significance as an important marker for paraptosis [8,165]. Its effective role in recruiting macrophages in inducing immunogenic cell death via paraptosis has also gained attention [7]. Thus, the involvement of HMGB-1 in both autophagy and paraptosis could indicate its importance as a major player in crosstalk. 

An overview of the central molecules mediating this crosstalk are outlined below in Table 2.

## 5. Crosstalk Between Autophagy & Paraptosis

Autophagy and paraptosis are two independent pathways, irrespective of their similarities concerning the proteins involved or the stimuli to which they are induced in different disease modes like cancer and neurodegeneration. The involvement of these pathways in the body helps in maintaining cellular homeostasis. Here, we point out that autophagy, a pro-survival mechanism, helps in removing damaged organelles and misfolded proteins, acting as a recycling machinery. Studies have reported that cancer cells exploit this survival mechanism to recover and continue to proliferate during therapy [24,50,167]. Predominantly, this pro-survival mechanism induces drug resistance and cancer relapse [82,100,168].

**Figure 3:**
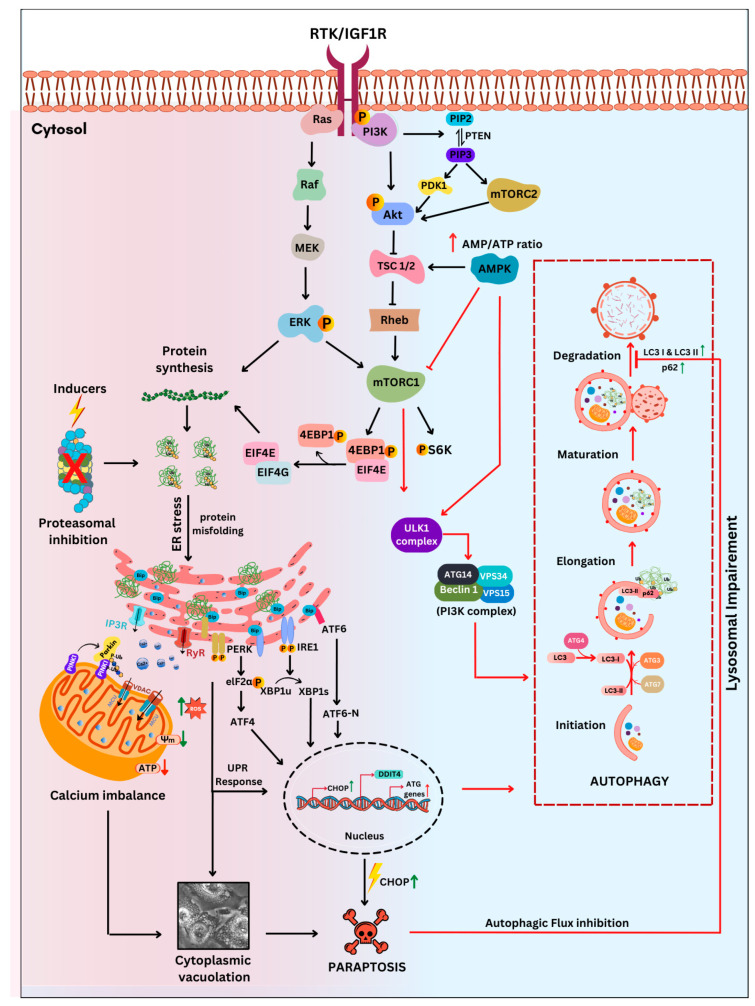
The intricate crosstalk between autophagy and paraptosis in cancer. The induction of the RTK/IGF1R receptor leads to the activation of MAPK and PI3K–Akt–mTORC1 signalling pathways. mTORC1 activation leads to protein synthesis. Increased translation and proteasomal inhibition by the inducer cause protein misfolding, leading to ER stress, disrupting Ca^2+^ homeostasis, ER, and mitochondrial function. Continued ER stress upregulates UPR-related proteins like PERK, IRE1α, and ATF6. PERK activation upregulates ATF4, leading to the induction of ATGs. At the same time, a decrease in translation due to UPR activates AMPK, which in turn inhibits mTORC1 and induces autophagy. Under higher ER stress conditions, ATF4 induces CHOP upregulation and inhibition of survival autophagy, leading to paraptotic cell death. Autophagy is marked with blue background and red arrows, paraptosis with pink background and black arrows, and crosstalk with a gradient background. RTK/IGF1R (receptor-tyrosine kinase/insulin-like growth factor 1 receptor); PI3K (phosphoinositide 3-kinase); PIP2 (phosphatidylinositol 4,5- bisphosphate); PTEN (phosphatase and tensin homologue); PIP3 (phosphatidylinositol 3,4,5-triphosphate); PDK1 (3-phosphoinositide-dependent protein kinase 1); AMPK (AMP-activated protein kinase); ULK1 (Unc 51-like autophagy-activating kinase); AKT (protein kinase B); TSC1/2 (tuberous sclerosis protein complex 1/2); Rheb (Ras homologue enriched in brain); mTORC1 (mammalian target of rapamycin complex 1); mTORC2 (mammalian target of rapamycin complex 2); S6K (S6 kinase); 4EBP1 (eIF4E-binding protein 1); EIF4E (eukaryotic initiation factor 4E); EIF4G (eukaryotic initiation factor 4G); VPS 34/15 (vacuolar protein sorting 34/15); ATGs (autophagy-related genes); IP3R (inositol 1,4,5-triphosphate receptors), RyR (ryanodine receptor); VDAC (voltage-dependent anion channel), MCU (mitochondria calcium uniporter); PINK1 (PTEN-induced putative kinase 1), PERK (protein kinase RNA-like ER kinase), IRE1α (inositol-requiring enzyme 1α); ATF6 (activating transcription factor 6); ATF4 (activating transcription factor 4); eIF2α (eukaryotic initiation factor 2α); XBP-1u (X box-binding protein 1 unspliced); XBP-1s (X box-binding protein 1 spliced); CHOP (C/EBP homologous protein); ROS (reactive oxygen species); DDIT4 (DNA damage-inducible transcript 4); LC3 (microtubule-associated light-chain protein); AMP (adenosine monophosphate); ATP (adenosine triphosphate).

Several studies report the activation of more than one mode of cell death, like paraptosis, apoptosis and autophagy [57,169,170], paraptosis, autophagy and ferroptosis [171], apoptosis and paraptosis [172,173], ferritinophagy and paraptosis [174], etc., during paraptosis in cancer cells, highlighting the crosstalk between them. Paraptosis is one among the alternate cell death pathways that is found to inhibit autophagy at a later stage [175]. Recently, natural compounds have been shown to induce paraptosis in various resistant cancers via targeting autophagy, as evidenced by increased accumulation of autophagy marker proteins [57], autophagosomes [25], and disruption of autophagic flux [22]. Moreover, studies have also reported that paraptosis can increase [49,85] or stay unaffected by autophagy inhibitors in cancer [72,174]. Additionally, one study reported that autophagy inhibitors attenuated δ-tocotrienol-induced [170] and Nur-77 binding 4-PQBH-induced [176] paraptosis, indicating the relationship between the two pathways to be complex and context-dependent. Based on the reported findings, we present a schematic in Figure 3, illustrating the crosstalk between paraptosis and autophagy in cancer.

Characteristic of most aggressive cancer cells is the overexpression of receptors like EGFR, FGFR, IGFR, and many growth factors [177]. Recent studies have indicated that paraptosis is activated via overexpression/hyperactivation of receptors like EGFR, IGFR, etc. [23]. As illustrated in Figure 3, receptor activation by hormones or growth factors leads to mTORC1 activation, which subsequently phosphorylates S6 kinase (S6K) and eukaryotic initiation factor (4E-BP1), leading to protein synthesis [39,66,178]. Several studies have demonstrated that MAPK activation and enhanced protein synthesis are critical for paraptosis induction [4,23,62,104]. However, some evidence suggests that taxol-induced paraptosis may occur independently of both protein synthesis and the MAPK pathway [179]. It has also been reported that many natural compounds or inducers of paraptosis bind to the newly synthesised proteins, promoting protein misfolding, concurrently inhibiting the proteasome, leading to proteasomal dysfunction [90]. During paraptosis, an altered proteasome, along with thioredoxin reductase inhibition, results in GSH depletion, thereby increasing proteotoxic stress in cancer cells [59,60,65]. This accumulation of misfolded proteins amplifies ER stress, eventually activating UPR [5,65]. During paraptosis, UPR activates the PERK pathway, which phosphorylates eIF2α, leading to the activation of ATF4 [75,77,180]. Downstream of PERK, ATF4 promotes the transcription of several autophagy-related genes like ATG3, ATG12, ATG5, and ATG7, as well as CHOP [94,181]. A recent study reported that VCP/p97 inhibition in HRas-mutated triple-negative breast cancer (TNBC) cells led to the activation of the ATF4–DDIT4 (ATF4–DNA damage-inducible transcript 4) axis, thereby increasing Akt signalling and translational recovery and enhanced proteotoxic stress-inducing paraptosis. Additionally, the knockdown of mTORC2/Rictor was found to suppress paraptosis, highlighting its regulatory role in this process [66]. Other ER sensors like IRE1α and ATF6 have also been reported to upregulate CHOP expression during UPR activation [23,182]. Further, when the UPR pathway is activated, it inhibits global protein translation [183]. In glioblastoma multiforme cells, the addition of mTOR inhibitors prevented NIM811-induced paraptosis by preserving autophagy and the UPR, thereby inhibiting cap-dependent translation [27]. Protein synthesis inhibition increases the AMP/ATP ratio, activating AMPK. Activated AMPK phosphorylates and inhibits mTORC1, enabling ULK activation, thereby initiating formation of the PI3K class III complex with the involvement of different autophagy-initiating targets like beclin 1, ATG14, VPS34, and VPS14 [184], leading to the initiation of autophagosome formation [185]. Except for reports on loss of beclin 1 during 8-p-hydroxybenzoyl tovarol-induced paraptosis [100] and ULK1 upregulation during NIM811-induced paraptosis [27], the change in the levels of other proteins like ATG14, VPS34, VPS14 in the initiation complex during paraptosis has not been probed. However, knockdown of ATG5 has been reported to sensitize glioblastoma cells to paraptosis [27]. UPR activation contributes to a decline in ATP levels. It was also reported that ATP synthesis and AMPK phosphorylation were reduced during δ-TT-mediated paraptosis via the disruption of calcium and increased ROS production [80]. The interplay between AMPK, mTORC1, and ULK1 pathways is necessary for cellular response to changes in nutrient deprivation or energy availability [183], but no studies have reported its role in regulating paraptosis. In addition, different compounds that affect mitochondrial integrity, which leads to mitophagy [186] and paraptosis [122], have been studied. The major regulators of mitophagy are PINK1 and parkin. PINK1 accumulates in the outer mitochondrial membrane of unhealthy mitochondria and recruits parkin [187]. The damaged mitochondria are marked by parkin, which promotes ubiquitination of mitochondria and removal by the autophagosomes. Interestingly, chalcomoracin has been reported to trigger paraptosis via mitophagy, suggesting a potential link between them, although their relationship remains unexplored [122]. Notably, several paraptosis inducers have been reported to disrupt mitochondria, resulting in swelling, a hallmark feature of paraptosis [5,188]. During paraptosis, calcium influx from the ER to mitochondria, along with ROS produced by natural products, contributes to a decrease in mitochondrial membrane potential (ΔΨm) [53,56,78,79,80,101,189]. This mitochondrial dysfunction further leads to increased ROS generation and impairs ATP synthesis, exacerbating cellular stress [80,190,191]. Concurrently, various studies have reported the upregulation of CHOP in directing cells towards cell death/paraptosis via cytoplasmic vacuolation [58,61,75,76]. This has been further proved by siRNA-mediated knockdown of CHOP [63,77,93]. Under conditions of severe ER stress and CHOP upregulation, survival autophagy, an adaptive response to counteract the stress conditions, gets inhibited mostly at the later stage of autophagosome and lysosome fusion, accompanied by accumulation of LC3I, LC3II, and p62. [21,22,23,25,192].

This shift from survival to death pathway underscores a potential therapeutic strategy for targeting cancer. Multiple factors contribute to this transition in a cell, including mutated receptors, a hyperactive MAPK pathway, elevated UPR response, proteasomal inhibition followed by misfolded protein accumulation, a decrease in GSH level, dysregulated Ca^2+^ homeostasis, elevated ER stress leading to increased proteotoxic stress by increased CHOP levels, inhibition of autophagic flux, and excess ROS-induced mitochondrial damage, and still more unexplored. Studies have reported that elevation of CHOP inhibits autophagy [193]. Given that CHOP has been shown to suppress autophagy and has been widely explored in paraptosis as an indicator of cellular stress, this can be a molecular switch that decides the occurrence of paraptosis when the apoptotic pathway is blocked. We tend to bring attention to the fact that paraptosis could be one of the emerging mechanisms that could target autophagy in cancer cells during therapy resistance. Thus, this crosstalk reflects the potential of paraptosis in targeting pro-survival mechanisms in cancer cells, highlighting the probability of an emerging therapeutic strategy for cancer.

## 6. Implications in Cancer Therapy

The complex interplay between autophagy and paraptosis can have therapeutic significance in cancer, with increasing chemoresistance to anti-apoptotic drugs. Many anticancer compounds have been known to induce paraptosis in cancer cells, including natural compounds [5] and metallic complexes [74,194]. The removal of autophagic substrates establishes cellular homeostasis in cancer cells. Therefore, understanding the change in autophagic flux (the rate of removal of autophagic substrates) will help in regulating autophagy and activating paraptosis. While various studies highlight the paraptotic form of cancer cell death, specific modifications in the cells’ autophagic machinery provide insights into the role of autophagy modulators in inducing paraptosis. In glioblastoma cells, Wang et al. (2017) reported the inhibition of survival autophagy by meIle4-cyclosporine (NIM811) during paraptosis induction and showed that the knockdown of a specific autophagy gene, such as beclin 1 or atg5, increased the susceptibility to paraptosis [27]. Similarly, studies with an autophagic inhibitor, elaiophylin, showed induction of paraptosis in ovarian cancer cells through activation of the MAPK pathway [23]. Another study on paraptosis-mediated hepatotoxicity with autophagy inhibition further suggests the active role of autophagy in non-apoptotic cell death [21]. It may be possible that the suppression of autophagy leads to the accumulation of misfolded proteins and ER stress, activating paraptosis. Selective autophagy induction by chalcomoracin leads to the activation of paraptosis by activating PINK1 [122], probably as a result of the constitutive deletion of functional mitochondria that causes cancer cell death, suggesting a dual role of autophagy in paraptosis-mediated cell death. There is potential for targeting the autophagy pathway for chemoresistant cancers, as it induces a non-apoptotic cell death pathway like paraptosis, but there are challenges in targeting these pathways in clinical settings due to tumour heterogeneity, specificity of modulators, and off-target effects [10]. Overcoming these challenges could provide a new avenue for cancer therapy.

The context-dependent balance between survival autophagy and paraptosis has significant implications for cancer therapy. The tumour microenvironment (TME) often exposes cancer cells to adverse stress conditions such as hypoxia and nutrient deprivation, initially promoting adaptive autophagy and enabling cancer cells to survive metabolic and proteotoxic stress and develop resistance to therapy [41]. Under mild ER stress, hypoxia promotes HIF-1α-dependent and AMPK-mediated autophagy, along with ER stress-induced adaptive responses, thereby supporting cellular survival [194]. However, during prolonged or severe hypoxia, protective autophagy becomes overwhelmed, leading to sustained ER stress, calcium dysregulation, and mitochondrial swelling—hallmark features of paraptosis. Nutrient deprivation can directly trigger autophagy through AMPK activation and mTOR inhibition, thereby facilitating metabolic recycling and survival under nutrient-deficient conditions [195]. Autophagy helps cancer cells develop drug resistance in the TME [41].

Combinatorial treatment of cancer cells with agents inducing paraptosis, like aloperine [22], jolkinolide–mTORi [196], and many others can address this problem in drug-resistant cancer cells, causing proteostasis collapse and organelle dysfunction, thus predisposing the cells towards paraptosis.

TME, through various mechanisms, serves as a safeguard for tumour cells. Secretion of growth factors such as VEGF and TGF-β promotes angiogenesis and immune evasion. Crosstalk between stromal and tumour cells activates signalling pathways such as PI3K–AKT, conferring resistance to apoptosis and therapy. Cancer-associated fibroblasts (CAFs), a major stromal component within the TME, contribute to drug resistance through various mechanisms, including extracellular matrix (ECM) remodelling, cytokine (IL-6, CXCL12) secretion, and inducing cancer stem cell formation, etc. [197]. The immune cells (Tregs) and myeloid-derived suppressor cells (MDSCs) within the TME reduce therapeutic efficacy mainly by creating an immunosuppressive niche [198]. A recent *in vivo* study reported that a paraptosis-inducing mixture of morusin–copper ions, when combined with an IDO inhibitor (NLG919), triggers both ICD and antitumour immunity by inhibiting regulatory T cells (Tregs) [7].

## 7. Preclinical and Clinical Studies on Modulators of Paraptosis and Autophagy

Although several natural compounds have been recognised as inducers of paraptosis, the therapeutic application of these molecules is yet to be explored. Identification of the molecular targets and pathways linked to this unique cell death mechanism can meet this research challenge in the future. While no compounds have proceeded to clinical trials, several paraptosis-inducing compounds have shown success in preclinical studies, which include epimedokoreanin B [25], elaiophylin [23], DHW-221 [73], and combinations such as celastrol–afatinib [192], paclitaxel–honokiol [194], mTORi and jolkinolide B [196], loperamide and bortezomib [199], bortezomib and nutlin 3 [200], bortezomib and ISRIB [201], vitamin B12b and DSFoxy [202], everolimus and ginsenoside Rh2 [26], lercanidipine and proteasome inhibitors [203], and radiation and chalcomoracin [204]. Cetylpyridinium chloride (CPC), as an FDA-approved drug primarily known for its antibacterial activity (UNII D9OM4SK49P), has been reported to induce paraptosis via severe ER stress in *in vivo* pancreatic ductal adenocarcinoma (PDAC) models, including patient-derived xenografts and orthotopic and genetically engineered PDAC mouse models, suggesting CPC as a promising candidate for clinical translation alone or in combinatorial therapy [94]. Paraptosis has significant therapeutic potential, particularly in overcoming drug resistance and sensitising cancer cells to conventional chemotherapy. Several paraptotic agents have demonstrated their efficacy in therapy-resistant cancer, as given in Table 3.

Numerous compounds, such as chloroquine [209,210,211], hydroxychloroquine [212,213], ULK1 inhibitors [92], VPS34 inhibitors [166], and ATG4 inhibitors [20], among others, have been identified as possible autophagy modulators. These substances, influencing autophagy by several different pathways, have shown promising results in preclinical and clinical research. Chloroquine and its hydroxyl analogue, hydroxychloroquine, clinically approved autophagic inhibitors, function by impairing the fusion of autophagosomes with lysosomes as a result of severe disorganisation of the Golgi and endo-lysosomal systems [214]. The success of *in vivo* studies using chloroquine and hydroxychloroquine provided the rationale for clinical trials for these compounds in combination with several compounds, thereby suggesting a potential anticancer therapy [215,216,217,218]. However, the specific targets of these compounds *in vivo*, as well as their dose-dependent effects, remain to be explored. Clinical studies associated with autophagy inhibitors are summarised in Table 4.

## 8. Current Limitations and Future Research Directions

Despite extensive studies on the role of autophagic modulators in cancer therapy resistance, we have not been successful in using autophagic regulators at all stages and types of cancer [243,244]. Paraptosis is a relatively less explored programmed cell death pathway, and many of its modulators have not yet been explored for cancer therapy [8]. Several natural products inducing paraptosis are identified, but they are specific to cancer cell types [5,178]. The co-occurrence of autophagy and paraptosis within cells has been observed in numerous instances during treatment with various natural products and photosensitizers [25,55,57,89,245]. However, there remains a gap in understanding the interplay between these pathways at the omics level, including transcriptomic, proteomic, and metabolomic levels. The major hallmark feature of paraptosis is the development of cytoplasmic vacuolation and dilation of ER and/or mitochondria [4,77]. To sustain cellular homeostasis, cancer cells require the breakdown of faulty ER and mitochondria [31]. However, limited research has delved into the specific targeting of organelles during paraptosis [183]. Investigating whether autophagy can shield cells from ER impairment that may trigger paraptosis presents an intriguing prospect. Studies have indicated that mitophagy plays a protective role in cancer cell demise by managing mitochondrial integrity, particularly in the context of apoptosis [246]. Further exploration is needed to understand the involvement of mitophagy and mitochondrial dynamics in the regulation of paraptosis.

Paraptosis is prevalent in rapidly proliferating cancer cells [48], preferably resistant to pro-apoptotic treatment [23,206]. Most of the chemotherapy for cancer is through the suppression of growth factor receptors or cell proliferation pathways. Targeted therapies against the components of MAPK pathways in BRAF-mutant and RAS-mutant cancers have been found to activate autophagy, leading to chemoresistance [247]. Mutations in several genes, including KRAS, PTEN, TP53, etc., have been found to be associated with different cancer types, triggering cell proliferation pathways like MAPK and PI3K–Akt–mTOR. KRAS mutation in PDAC triggers cell proliferation through MAPK activation. This helps PDAC cells to undergo paraptosis when treated with CPC [94]. Similarly, PTEN loss in acute pro-myelocytic leukaemia leads to hyperactivation of mTOR, making cells vulnerable to paraptosis when exposed to honokiol [72]. A recent report highlighted an association between TP53 mutations and paraptosis in gastric cancer patients, identifying TP53 as one of the paraptosis-related genes [248]. In this scenario, induction of paraptosis could be a promising strategy, particularly in highly proliferative cancer cells. Compounds showing promising effects at the preclinical level could be further evaluated in clinical trials. Understanding the unique regulatory role of ATF4 in the interplay between autophagy and paraptosis will be interesting, given its dual role in inducing paraptosis and autophagy. Investigating the mechanism regulating the switch between these pathways holds promise. A deeper understanding of the crosstalk between autophagy and paraptosis can be achieved through preclinical studies of ATG-knockout mouse models. Successful findings can then be clinically tested for possible translation. The late-stage inhibition of autophagy during paraptosis might be one of the many regulatory mechanisms that should be focused on [22,23,191]. Identifying autophagic inhibitors having the ability to induce paraptosis in cancer cells can be a potential candidate for cancer therapy. The application of paraptosis-inducing agents and autophagic inhibitors can represent a more effective therapeutic strategy for cancer treatment.

Autophagy and paraptosis, attractive targets for modern cancer therapy, possess their own conceptual and translational limitations. Inducing paraptosis might offer a potential solution to chemoresistance, but its therapeutic application remains a challenge, as there are no universal markers for paraptosis besides morphological hallmark features. Studies focusing on potential markers and signalling pathways for paraptosis might offer a promising solution in this area. Despite extensive studies on the role of autophagic modulators in cancer therapy resistance, chemoresistance cannot always be attributed solely to autophagy inhibition [244,245]. Cancer-resistant cells exhibit various autophagy states, ranging from survival autophagy, promoting cell survival, to antitumor activity contributing to cell death [166]. Though multiple preclinical studies show promising effects of paraptosis in chemoresistant cancers, studies involving both autophagy and paraptosis are currently limited to in vitro systems. The co-occurrence of autophagy and paraptosis within cells has been observed in numerous instances during treatment with various natural products and photosensitizers in vitro [25,55,57,100,246]. While these results provide valuable insights, understanding the roles of a complex tumour microenvironment, systemic interactions, and dosage response in this type of treatment becomes imperative. Animal studies can help fill the gaps in in vitro studies. Studying autophagy gene knockouts in paraptosis can help determine whether autophagy is essential for initiation, progression, or suppression of paraptosis and the physiological or pathological relevance in animals. This can help in better understanding the relationship between the two processes and provide a solid foundation for possible clinical translation.

With growing insights into immune evasion mechanisms, tumour immunotherapy has been developed to harness or augment the immune system to effectively target or suppress tumours. Autophagy plays a dual role in cancer immunity, either enhancing antitumour responses by promoting T-cell function and antigen presentation or contributing to immune evasion and drug resistance by supporting tumour cell survival [248]. Autophagy also plays a cytoprotective role in cancer via transient activation of PERK signalling to relieve stress conditions [89]. Interestingly, a study has highlighted the knockout of PERK, triggering Sec61β-induced paraptosis in melanoma cancer. Excessive ER stress in a PERK^KO^ tumour resulted in reduced p-eIF2α levels and production of type 1 interferon (IFN-1) by dendritic cells. This was accompanied by the orchestrated release of several DAMPs, such as HMGB1 and ATP, leading to immunogenic cell death (ICD) via paraptosis [6]. The antitumour potential of paraptosis may also modulate autophagy to enhance immune responses against tumour cells through the induction of immunogenic cell death.

Emerging evidence demonstrates a crucial role of paraptosis in modulating tumour progression, prognosis, and the immune microenvironment across multiple cancer types, including breast [249], gastric [248,250], glioma [251], and lung adenocarcinoma [252]. Studies have correlated the discovery of paraptosis-related gene signatures with robust patient classification and development of the paraptosis-related risk score (PRRS), which predicts survival outcomes, immune infiltration, and drug sensitivity [249]. A low PRRS is associated with a favourable prognosis and enhanced antitumor immunity, whereas a high PRRS reflects poor clinical outcomes and therapeutic resistance [252]. Collectively, future studies should focus on elucidating the molecular mechanisms through which key paraptosis-related genes, such as CDKN3 [253], regulate paraptotic cell death. Prospective validation of PRRS in large-scale, multicenter cohorts will strengthen its clinical applicability. Moreover, correlating paraptosis signatures with other programmed cell death pathways via single-cell sequencing and transcriptomic approaches may further enhance prognostic accuracy and therapeutic implications. Overall, targeting paraptosis-related genes represents a promising direction for biomarker identification and in advancing personalised cancer therapy and improving patient outcomes.

## 9. Conclusions

With the rise in incidence of chemoresistance, there is a pressing need for the development of therapies to combat cancers. Recent developments in the study of non-apoptotic cell death pathways and a comprehensive understanding of the function of autophagy in cancer kindled our interest in the relationship between these processes. Several findings linked the involvement of autophagy inhibition with the induction of paraptosis, proving their antagonistic nature. Further, with the findings of the key molecules playing a significant role in both processes, it becomes easier to target such molecules to initiate cancer cell death. Thus, investigating the dynamic interplay between autophagy and paraptosis is crucial for cancer therapy and other pathophysiological conditions like viral infections and neurodegeneration.

## Figures and Tables

**Table 1:** Distinguishing features of autophagy and paraptosis.

Distinguishing Features	Autophagy	Paraptosis
Characteristics	Formation of autophagosomal vacuoles [20,29] Increased activation of cargo receptors [31] Autophagosome-lysosome fusion [29] Increased lysosomal activity [29]	Extensive cytoplasmic vacuolation due to swelling of the ER and mitochondria [4] Caspase-independent cell death [4] Protein misfolding and ER stress [48] Disruption of sulfhydryl Homeostasis [84] Calcium imbalance [56] ROS generation [85]
Vacuoles	Double-membraned vacuoles containing aggregated proteins and dysfunctional organelles [20,29]	Single-membraned empty vacuoles [86]
Inducers/Stimuli	Nutrient stress or hormonal induction [87] Organelle/DNA damage [38] Hypoxia [38] Rapamycin, or drug-induced ER stress [88,89]	Natural products [90] Metallic complexes [53] Cancer drugs and phytochemicals [90] Nanodrugs [54] Photosensitizers [55] Ion channel activators [61]
Inhibitors	PI3K class III inhibitors 3-methyladenine (3MA), wortmannin, LY294002, PT210 GSK-2126458 [91] VPS34 inhibitors Spautin 1, SAR405, VPS34-IN1, PIK-III [91] ULK inhibitors MRT68921, SBI-0206965 [92] Lysosome inhibitors Pepstatin A, E64d, bafilomycin A, chloroquine, hydroxychloroquine, Lys05, ARN5187, clomipramine, lucanthone [91]	Protein synthesis inhibitor cycloheximide (CHX) [85] ER stress inhibitor 4-phenylbutyric acid (4PBA) [93] PTPN11, KRAS, MEK inhibitors SHP099, BAY293, UO126 [23] ROS scavengers GSH, N-acetyl cysteine (NAC) [85] Cytosolic Ca^2+^ chelator BAPTA-AM [78] p38 inhibitors Doramapimod; SB203580 [77,94]

**Table 2:** Key proteins regulating the intersection of autophagy and paraptosis.

Sl. No:	Major Players	Autophagy	Paraptosis
1	Beclin 1	Beclin 1, a part of the PI3K-class III complex, has a role in nucleation [16]	Loss of beclin 1 leads to ER-stress-induced paraptosis [100]
2	MAP1LC3B	LC3B plays a major role in autophagosome formation after cleavage and lipidation. During autophagy, the level of LC3-II increases [87]	During paraptosis, both LC3-I and LC3-II levels increase in the cells. The specific role of LC3B in paraptosis is not known [48,49]
3	SQSTM1/p62	p62 plays a key role in the sequestration of ubiquitinated proteins inside the autophagosome. The level of p62 decreases during autophagy [31]	p62 binds to ubiquitinated proteins and accumulates inside the cell [48,49]
4	NBR1	NBR1 function is similar to p62, and its level is decreased during autophagy [109]	NBR1 function is similar to p62, and its level is increased during paraptosis [113]
5	PINK1	Vital inducer of mitophagy [121]	During chalcomoracin-induced paraptosis, PINK1 is upregulated. PINK1-dependent mitophagy induces paraptosis [122]
6	Alix	Interaction of the ATG3– ATG12 complex with Alix promotes the maturation of autophagosomes [124]	Alix acts as an endogenous inhibitor of paraptosis [62]
7	Bip	Chaperone protein binds to ubiquitinated polypeptides, activates UPR, and relieves stress in the ER. Plays an important role in stress- induced autophagy [141]	Bip level is increased during paraptosis with increased accumulation of misfolded proteins leading to UPR [58,75,82]
8	PERK	PERK mediates UPR and activates autophagy [137]	Activation of PERK in cancer cells induces paraptosis [159]
9	IRE1α	IRE1α regulates autophagy by mediating UPR [137]	Activation of IRE1α induces paraptosis through UPR [159]
10	ATF6	ATF6 activation promotes UPR and autophagy [137]	Upregulation of ATF6 is found during paraptosis [159]
11	CHOP/GADD153	CHOP is linked to both cell death and autophagy. Depending upon the severity of ER stress, the cell fate is decided [141]	Increased ER stress initiated UPR response, thereby increasing ATF4 and CHOP which leads to activation of paraptosis [23,68]
12	USP10	USP10, a deubiquitinase, is shown to prevent beclin 1 degradation and induce autophagy [162]	USP10 downregulation inhibits paraptosis [63]
13	Calcium channel receptors	An imbalance of Ca^2+^ influx can cause survival autophagy [149]	Change in Ca^2+^ homeostasis leading to mitochondrial Ca^2+^ overload can cause paraptosis [112,150]
14	CDKIs	Cell cycle arrest during autophagy inhibits tumour growth. Upregulation of CDKIs like p21 and p27 occurs during autophagy in cancer [154,155]	Cell cycle arrest is observed during paraptosis in cancer cells. CDKIs like p21 and p27 are found to be upregulated during paraptosis, leading to cell cycle arrest [22]
15	mTORC1	Inactive form involved in the initiation of autophagy [30,70]	Active form involved in the initiation of paraptosis [23,66,72]
16	MAPK	Inhibition of MAPK signalling inactivates mTORC1 during autophagy [57]	Activation of MAPK leads to paraptosis through increased cell proliferation and translation [4,23,62,94]
17	RTK/IGF1R/ EGFR	IGF1R depletion activates autophagy through inhibition of mTORC1 [166]	Activation of the IGF1R receptor leads to the induction of paraptosis [4]
18	TRIB3	Interrupting the TRIB3-p62 interaction activates autophagic flux [114]	c-Myc mediated accumulation of TRIB3/p62+ aggresome triggering paraptosis [26]
19	HMGB-1	HMGB-1 activates the beclin 1–PI3K-III complex in the cytoplasm to promote autophagy [163,164]	Translocation of HMGB-1 from the nucleus to the cell periphery is found during paraptosis [163]

**Table 3:** Preclinical studies on paraptosis-inducing agents showing efficacy against drug-resistant cancer models.

Sl. No	Treatment Inducing Paraptosis	Chemical Structure	Cancer Models	Resistant to Drug (s)	Mechanism of Action	Reference
1	Elaiophylin	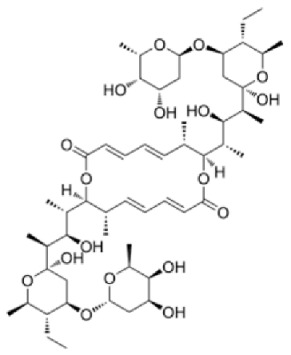	Ovarian cancer	Platinum, taxane, or PARPi	SHP2/SOS/MAPK signalling and autophagy inhibition	[23]
2	α-Hederin	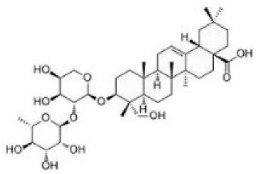	Colorectal cancer	5-fluorouracil	GPCR-mediated PLCβ3/IP3R/Ca^2+^/PKCα signalling	[205]
3	CPYPP and curcumin	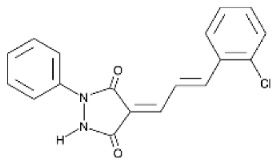 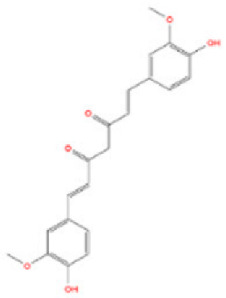	Head and neck cancer	Docetaxel	Nuclear stress- induced activation of CDK7/CDK9–Rpb1 driving transcriptional regulation	[159]
4	DHW-221	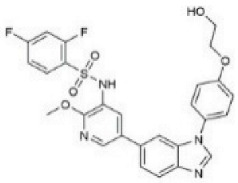	Lung adenocarcinoma	Taxol	Elevated ER stress MAPK signalling	[73]
5	Thioxotriazole copper (II) complex A0, A0 (cis- [CuCl_2_(H_2_L)]Cl) copper (II) complex of HL (4-amino-1,4-dihidro-3-(2-pyridyl)-5-thioxo-1,2,4-triazole)	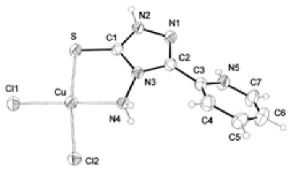	Fibrosarcoma	Cisplatin	UPR activation leading to ER stress	[75]
6	Monocationic hydrophilic complexes [Cu(thp)_4_]^+^3	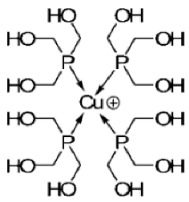	Colon cancer	Doxorubicin	Lysosomal damage mediated by an increase in ROS, causing a decrease in MMP	[206]
7	Phosphine copper(I) complex [Cu(thp)_4_] [PF6] (CP)	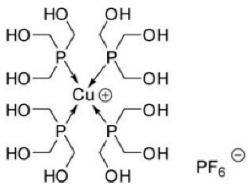	Colon cancer	Platinum	Proteasome inhibition leading to ER stress	[93]
8	Aloperine	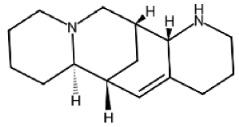	Glioblastoma	Temozolomide	Inhibition of late-stage autophagy	[22]
9	Bipyridine-Silver(I) Compound	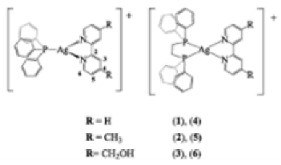	Ovarian cancer and colon carcinoma	Carboplatin/ oxaliplatin	Elevated ER stress and MAPK signalling accompanied by mitochondrial dysfunction	[207]
10	FAK siRNA-loaded exosomes (CT-Exo-siFAK1)	-	Colon cancer	Cetuximab	Elevated ER stress	[208]
11	Flemiphilippinin A	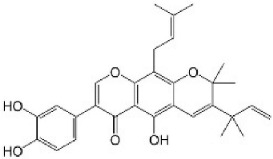	Lung adenocarcinoma	Gefitinib	c-Myc-mediated ER stress and mitochondrial dysfunction	[190]
12	Jolkinolide B Jolkinolide B + mTORi	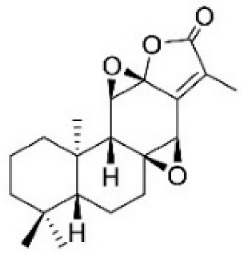	Bladder cancer Bladder cancer	Cisplatin Cisplatin	Elevated ER stress and an increase in ROS production due to TrxR1 inhibition and GSH depletion Dual inhibition of Akt feedback activation and survival autophagy	[60,196]
13	Honokiol	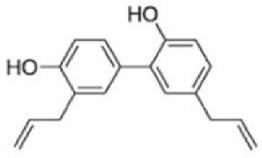	Non-small-cell lung cancer	Paclitaxel	Proteasomal inhibition mediated ER stress, Ca^2+^ overload, leading to mitochondrial dysfunction	[194]
14	Celastrol	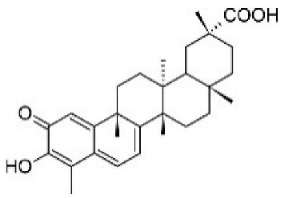	Non-small-cell lung cancer	Afatinib	ER stress, UPR activation, ROS accumulation, and Ca^2+^ overload	[192]
15	Fangchinoline	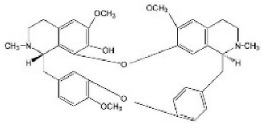	Renal cell carcinoma	Paclitaxel	Elevated ER stress, ROS accumulation	[59]
16	Disulfiram	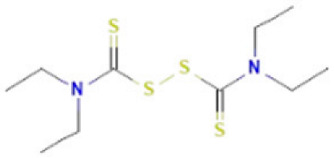	Prostate cancer	Cabazitaxel	ER stress and ROS generation	[54]
17	Loperamide	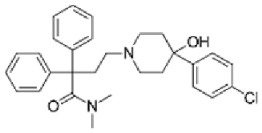	Colon cancer	Bortezomib	ER stress, Ca^2+^ imbalance and CHOP upregulation	[199]
18	Nutlin 3	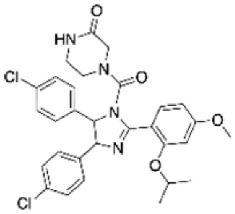	Breast cancer, glioblastoma, colon and cervical cancer	Bortezomib	ER stress, CHOP upregulation, and disruption of Ca^2+^ homeostasis	[200]
19	ISRIB	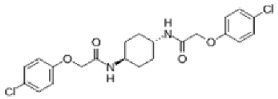	Breast cancer	Bortezomib	Enhanced proteotoxic stress	[201]
20	Lercanidipine	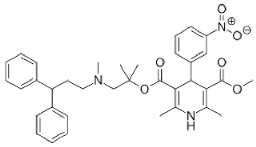	Breast cancer and hepatocellular carcinoma	Bortezomib, Carfilzomib, and Ixazomib	Enhanced ER stress and mitochondrial Ca^2+^ overload	[203]
21	Ginsenoside Rh2 and everolimus	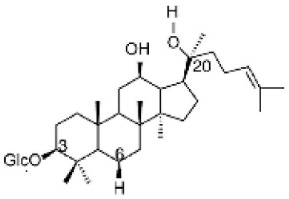 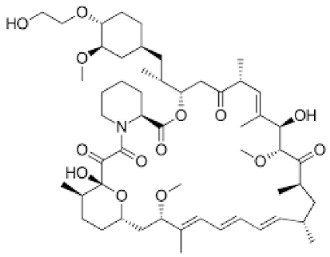	EGFR-mutant lung cancer	Osimertinib	c-MYC mediated the accumulation of TRIB3/P62+ aggresomes	[26]

**Table 4:** Clinical studies with autophagy inhibitors chloroquine (CQ) and hydroxychloroquine (HCQ).

Clinical Trial	Cancer	Phase	References
CQ	Breast	II	[219]
	Breast	I	[220]
	Brain metastases	I	[221]
CQ+ Taxane	Breast	II	[222]
CQ/HCQ+ Carboplatin, Gemcitabine	Advanced solid tumours	I	[223]
CQ+ Gemcitabine	Metastatic or unresectable pancreatic cancer	I	[224]
CQ+ Bortezomib, Cyclophosphamide	Relapsed and refractory multiple myeloma	II	[225]
CQ+ Temozolomide	Glioblastoma	Ib	[226]
CQ+ Carmustine	Glioblastoma	I	[227]
CQ+ Metformin	Solid tumours (IDH1 mutations)	Ib	[228]
HCQ	Metastatic pancreatic adenocarcinoma	II	[229]
HCQ+ Aldesleukin	Metastatic renal cell carcinoma	II	[230]
HCQ+ Carboplatin, Paclitaxel, Bevacizumab	Non-small-cell lung cancer	Ib/II	[231]
HCQ+ Vorinostat	Metastatic colorectal cancer	II	[232]
HCQ+ Regorafenib, Entinostat	Metastatic colorectal cancer	I	[233]
HCQ+ Carboplatin, Paclitaxel, Bevacizumab	Non-small-cell lung cancer (KRAS mutations)	Ib	[234]
HCQ+ Temozolomide	Advanced solid tumours and melanoma	I	[216]
HCQ+ FOLFOX/ Bevacizumab	Advanced colorectal cancer	II	[235]
HCQ+ Gemcitabine	Pancreatic adenocarcinoma	I/II	[236]
HCQ+ Gemcitabine/Nab-Paclitaxel	Advanced pancreatic cancer	II	[237]
HCQ+ Erlotinib	Advanced non-small-cell lung cancer	I	[238]
HCQ+ Temozolomide	Glioblastoma	I/II	[217]
HCQ+ Bortezomib	Multiple myeloma	I	[239]
HCQ+ Cyclophosphamide, Rapamycin	Relapsed or refractory multiple myeloma	I	[88]
HCQ+ Sirolimus	Lymphangioleiomyomatosis	I	[240]
HCQ+ Everolimus	Renal cell carcinoma	I/II	[241]
HCQ+ Gemcitabine/ Nab-Paclitaxel	Pancreatic cancer	II	[242]

## Data Availability

No new data were created or analyzed in this study.

## Abbreviations

The following abbreviations are used in this manuscript.

ATF4	activating transcription factor 4
ATF6	activating transcription factor 6
AMPK	AMP-activated protein kinase
ATG	autophagy-related gene
BECN1	beclin 1
CHOP	C/EBP homologous protein
DAMPs	damage-associated molecular patterns
DDIT4	DNA damage-inducible transcript 4
ERAD	ER-associated degradation
FAK	focal adhesion kinase
GPCR	G protein-coupled receptor
IGF1R	insulin-like growth factor 1 receptor
IP3R	inositol 1,4,5-triphosphate receptor
IRE1	inositol-requiring enzyme 1
KRAS	Kirsten rat sarcoma viral oncogene homologue
MAPK	mitogen-activated protein kinase
mTOR	mammalian target of rapamycin
PARPi	poly(ADP-ribose) polymerase inhibitor
PERK	PKR-like ER kinase
PTPN11	protein tyrosine phosphatase nonreceptor type 11
RTK	receptor tyrosine kinase
RyR	ryanodine receptor
SERCAs	sarco/endoplasmic Ca^2+^-ATPases
SHP2	Src homology 2-containing protein tyrosine phosphatase 2
SOS	Son of Sevenless
TRIP13	thyroid hormone receptor-interacting protein 13
TrxR1	thioredoxin reductase 1
ULK	Unc 51-like kinase
VCP	valosin-containing protein
VDAC	voltage-dependent anion channel
VPS34/15	vacuolar protein sorting 34/15
